# The Impact of Type 2 Diabetes in Parkinson's Disease

**DOI:** 10.1002/mds.29122

**Published:** 2022-06-14

**Authors:** Dilan Athauda, James Evans, Anna Wernick, Gurvir Virdi, Minee L. Choi, Michael Lawton, Nirosen Vijiaratnam, Christine Girges, Yoav Ben‐Shlomo, Khalida Ismail, Huw Morris, Donald Grosset, Thomas Foltynie, Sonia Gandhi

**Affiliations:** ^1^ Neurodegeneration Biology Laboratory Francis Crick Institute London United Kingdom; ^2^ UCL Queen Square Institute of Neurology London United Kingdom; ^3^ Department of Clinical and Movement Neurosciences UCL Queen Square Institute of Neurology London United Kingdom; ^4^ School of Social and Community Medicine University of Bristol Bristol United Kingdom; ^5^ Department of Psychological Medicine King's College London United Kingdom; ^6^ Institute of Neurological Sciences Queen Elizabeth University Hospital Glasgow United Kingdom

**Keywords:** Parkinson's, type 2 diabetes, disease progression, cognitive impairment

## Abstract

**Background:**

Type 2 diabetes (T2DM) is an established risk factor for developing Parkinson's disease (PD), but its effect on disease progression is not well understood.

**Objective:**

The aim of this study was to investigate the influence of T2DM on aspects of disease progression in PD.

**Methods:**

We analyzed data from the Tracking Parkinson's study to examine the effects of comorbid T2DM on PD progression and quality of life by comparing symptom severity scores assessing a range of motor and nonmotor symptoms.

**Results:**

We identified 167 (8.7%) patients with PD and T2DM (PD + T2DM) and 1763 (91.3%) patients with PD without T2DM (PD). After controlling for confounders, patients with T2DM had more severe motor symptoms, as assessed by Movement Disorder Society Unified Parkinson's Disease Rating Scale, Part III (25.8 [0.9] vs. 22.5 [0.3] *P* = 0.002), and nonmotor symptoms, as assessed by Non‐Motor Symptoms Scale total (38.4 [2.5] vs. 31.8 [0.7] *P* < 0.001), and were significantly more likely to report loss of independence (odds ratio, 2.08; 95% confidence interval [CI]: 1.34–3.25; *P* = 0.001) and depression (odds ratio, 1.62; CI: 1.10–2.39; *P* = 0.015). Furthermore, over time, patients with T2DM had significantly faster motor symptom progression (*P* = 0.012), developed worse mood symptoms (*P* = 0.041), and were more likely to develop substantial gait impairment (hazard ratio, 1.55; CI: 1.07–2.23; *P* = 0.020) and mild cognitive impairment (hazard ratio, 1.7; CI: 1.24–2.51; *P* = 0.002) compared with the PD group.

**Conclusions:**

In the largest study to date, T2DM is associated with faster disease progression in Parkinson's, highlighting an interaction between these two diseases. Because it is a potentially modifiable metabolic state, with multiple peripheral and central targets for intervention, it may represent a target for alleviating parkinsonian symptoms and slowing progression to disability and dementia. © 2022 The Authors. *Movement Disorders* published by Wiley Periodicals LLC on behalf of International Parkinson and Movement Disorder Society

## Graphical abstract

Comorbid type 2 diabetes (T2DM) is associated with more severe Parkinson's disease (PD), affecting motor symptoms, nonmotor symptoms, and cognition. Patients with T2DM develop substantial gait impairment and mild cognitive impairment faster than those without T2DM. Targeting insulin resistance in PD may offer a therapeutic window in which to halt further PD progression.

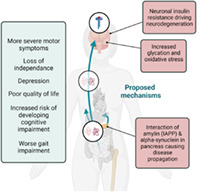

Parkinson's disease (PD) affects 6 million people worldwide, and its prevalence is expected to increase in response to an aging population.^1^ Although aging is undoubtedly the most important risk factor, there is evidence that type 2 diabetes (T2DM) is a modest risk factor.^2^, ^3^ In addition, accumulating evidence suggests these diseases share common biological mechanisms (reviewed by Santiago and Potashkin^4^) and share genetic links,^5^ highlighting dysfunctional insulin signaling as a possible convergent pathway responsible for the association between these conditions.^6^ Insulin receptors are widely expressed throughout the brain, and evidence of defective neuronal insulin signaling has been found in postmortem studies in PD,^7^, ^8^ multiple system atrophy, and Alzheimer's disease (AD).^9^ Defective insulin signaling leads to dysfunction of major downstream pathways, including the Akt and mitogen‐activated protein kinase pathways. These in turn regulate a variety of processes essential for maintaining neuronal survival, including autophagy, protein synthesis, inflammation, nerve cell metabolism, and dopamine synthesis and clearance, all of which become disrupted in PD and thus may be exacerbated/triggered by comorbid T2DM. PD + T2DM patients exhibit more severe dopaminergic depletion in the caudate and ventral striatum^10^, ^11^ and more frontotemporal cortical atrophy compared with those without T2DM,^12^, ^13^ further supporting the influence of insulin resistance on neurodegeneration.

Beyond its effects on PD risk, a handful of studies have evaluated the influence of T2DM on disease progression and suggested comorbid T2DM may be associated with faster motor^11^, ^14^ and cognitive decline^15^ in comparison with patients with PD without T2DM. However, generalizability from these studies has been limited by the relatively small numbers of patients included (Supporting Information Table S1).

Using the Tracking Parkinson's cohort, a large long‐term observational study into PD, our aims were, first, to evaluate the association of comorbid T2DM on PD severity in patients recently diagnosed with PD and, second, to determine whether T2DM negatively affects disease progression. In view of accumulating data suggesting antiglycemic treatments may be useful in the treatment of PD, we also conducted an exploratory analysis for our third aim: to determine whether metformin use in patients with diabetes confers any protective effects on the severity and long‐term outcomes.

## Subjects and Methods

### Study Design and Data Collection

Data from Tracking Parkinson's, including demographic, clinical, imaging, and biospecimen measures, that have been collected for more than 6 years were analyzed. The study setup and design have been previously reported.^16^ Enrolled participants were recruited with a clinical diagnosis of PD fulfilling UK Brain Bank criteria and included both drug‐naive and treated patients aged 18–90 years. Recent‐onset cases were diagnosed with PD in the preceding 3.5 years, and recruitment was completed between February 2012 and May 2014. Seventy‐two sites across the United Kingdom providing secondary care treatment for patients with PD as part of the UK National Health Service participated, and visits occurred every 6 months, with repeated observations and blood sampling every 18 months. The following features were collected: demographics, diagnostic features at presentation, ethnicity, education, medication history, body mass index (BMI), and comorbidities. A concurrent diagnosis of preexisting T2DM was based on self‐report at baseline. Levodopa equivalent daily dose (LEDD) was calculated using an established formula.^17^

### Outcomes

Due to previous evidence that insulin resistance may impact several aspects of PD progression via multiple molecular mechanisms, we have chosen a number of motor and nonmotor outcomes that could highlight the influence of T2DM on PD. We used several motor and nonmotor features previously established as “severe/advanced” disease markers or clinical milestones,^18^ and we used them to identify their appearance at baseline entry into the study (the reasoning that, even at a short disease duration, patients with a greater number of these markers at baseline could be identified as having a more severe disease phenotype), and also used them to monitor long‐term disease progression.

Appearances of motor fluctuations, dyskinesias, impulse control disorders (ICDs), dopamine dysregulation, and hallucinations were regarded as binary endpoints, corresponding to Movement Disorder Society Unified Parkinson's Disease Rating Scale (MDS‐UPDRS) item responses of ≥1.^19^ Onset of cognitive decline was determined using MDS level I criteria^20^ to define mild cognitive impairment (MCI), and we used Montreal Cognitive Assessment (MoCA) test scores to categorize cases into normal (MoCA ≥ 26) and MCI (MoCA 21–25, plus no functional cognitive impairment as assessed by MDS‐UPDRS 1.1).^21^, ^22^ Substantial gait impairment was defined as score >3 on MDS‐UPDRS Part III question 10. The Non‐Motor Symptoms Scale (NMSS) was used to derive nonmotor symptom burden. Loss of independence is an important determinant of quality of life^23^ and has been defined previously as Hoehn & Yahr (H&Y) stage >3^24^ and Schwab and England Activities of Daily Living scale <80%.^25^ The Questionnaire for Impulsive‐Compulsive Disorders in PD (QUIP) was used to define ICDs and impulse control and related disorders (ICD‐RDs). Individuals with ICD were defined as any affirmative response to questions pertaining to pathological gambling, hypersexuality, binge eating, and compulsive buying, and ICD‐RDs as individuals with any affirmative response to questions regarding punding, hoarding, and walkabout/aimless wandering.^26^ Depression was defined using the Leeds Anxiety and Depression Scale with a cutoff score >6.^27^ To conduct our exploratory analysis, we used medication lists to identify patients treated with antidiabetic medication. Metformin was the most commonly prescribed drug, which directed our exploratory analysis to compare these patients with patients not on metformin; however, due to insufficient numbers of patients prescribed glucagon‐like peptide‐1 drugs, we were unable to perform this analysis.

### Statistical Analyses

Group comparisons between PD and PD + T2DM groups were performed at baseline, using multivariate analysis of covariance with post hoc Bonferroni correction for multiple comparisons. Categorical variables were compared using Fisher's exact test, and multivariate logistic regression was used to determine the adjusted odds ratios (ORs). Potential confounders were included in the statistical models guided by mechanisms proposed and depicted in directed acyclic graphs, which considers each variable in relation to the exposure and outcome (Supporting Information Fig. S1), because both the failure to adjust for a confounder and overadjusting for an intermediate variable can lead to biased results.^28^ This highlighted age, sex, ethnicity, and BMI as covariates. We also included PD duration, vascular score, H&Y stage, and LEDD as additional covariates. Vascular score was determined by the number of vascular diseases (angina, heart failure, stroke, heart attack, etc.) and patients categorized as having none, one, or two or more diseases.

For longitudinal analysis, we performed a survival analysis to determine whether T2DM influenced PD progression based on the appearance of previously defined clinical milestones. Kaplan–Meier survival curves were plotted, and log‐rank tests were performed. Analyses were repeated, including age, sex, ethnicity, PD duration, vascular score, baseline H&Y stage, LEDD, and BMI as covariates. Only the time to occurrence of the first event in a category for a given subject was used in the subsequent Cox regression model. Participants at baseline who had already developed the clinical milestone (outcome) were excluded from the model.

To assess the effect of T2DM on rate of change of a given symptom (eg, MDS‐UPDRS Part III score), we used separate linear mixed effects models with robust variance estimates, with examination of the interaction effects of group (PD vs. T2DM) and time. Symptom progression was modeled adjusting for age, sex, vascular score, disease duration, ethnicity, baseline LEDD, and the baseline variable value as fixed effects for the intercept and slope. Participant‐specific random effects were included as both a random intercept and a random slope to account for the correlation in repeated measurements within the same participant. Due to the increasing number of patient dropouts over the follow‐up period, we chose a cutoff of 50% missing data as the upper threshold, and thus chose visit 3 as the “endpoint” of the study, representing a mean follow‐up time of 37.8 (SD 4.3) months since study entry. Analyses were performed using SPSS statistical software, version 26.0 (IBM Corp., Armonk, NY). All *P* values presented have been adjusted for multiple comparisons.

An additional sensitivity analysis was conducted using vascular score as the independent variable to evaluate whether increased vascular disease is the driver of T2DM‐mediated effects on disease progression and allowed novel pathophysiological insights to be made. Patients were categorized into patients with PD with no T2DM and no vascular diseases (PD/VasR), patients with PD with no T2DM but with one or more vascular diseases (PD/VasR^+^), and patients with comorbid T2DM (PD + T2DM).

## Results

### Characteristics of Cohort at Baseline

**FIG 1:**
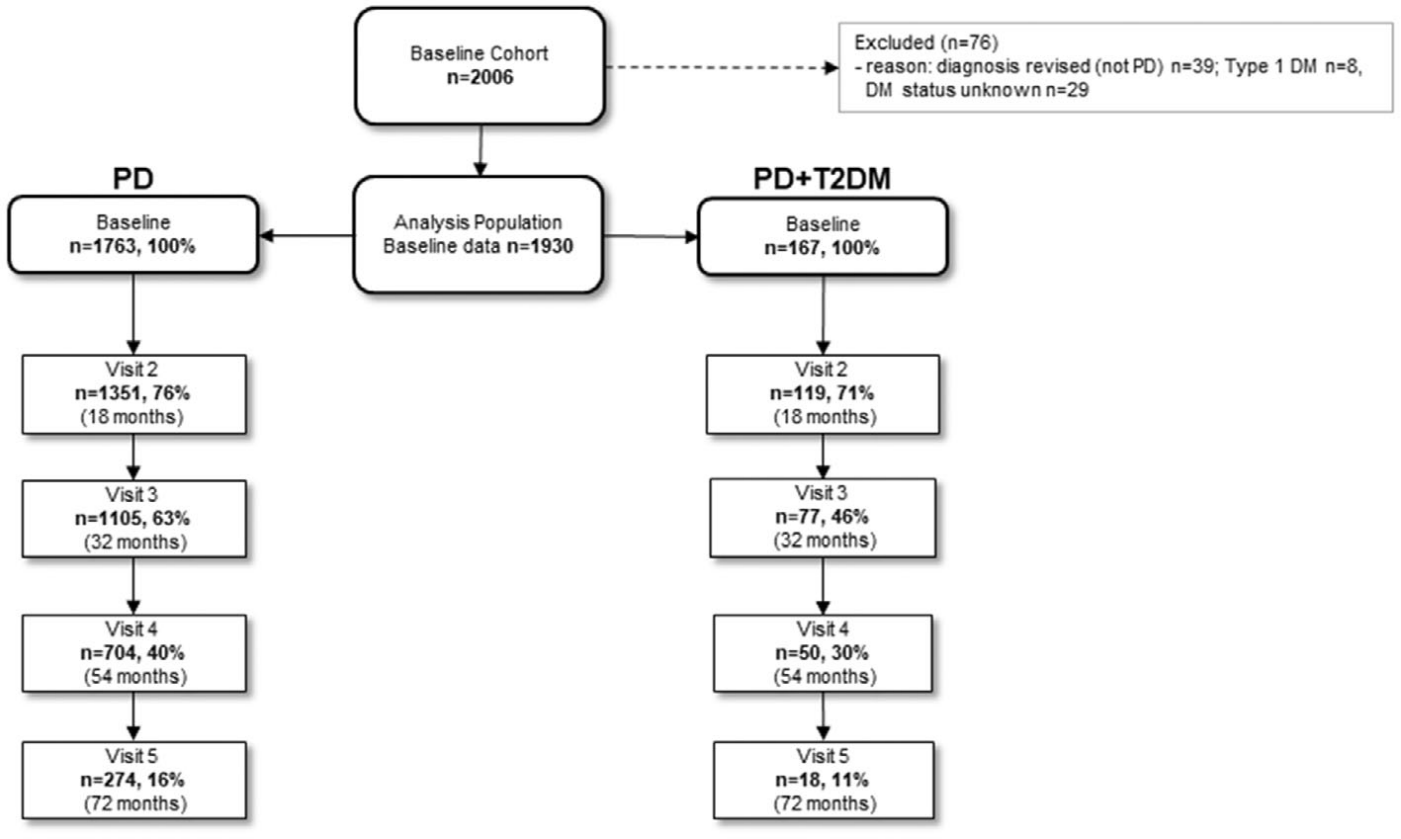
Flow diagram of the study population. DM, diabetes mellitus; PD, Parkinson's disease; T2DM, type 2 diabetes mellitus.

The main analysis group consisted of 1930 individuals, of whom 167 (8.7%) had comorbid T2DM (PD + T2DM) and 1763 (91.3%) did not (PD) (Fig. 1). Demographic features and clinical features are summarized in Table 1.

**TABLE 1:** Early clinical features in PD cases without T2DM, compared with cases with T2DM

Demographics	PD (n = 1763)^a^	PD + T2DM (n = 167)^a^	*P* value
Age (y), mean (SE)	67.2 (0.2)	71.1 (0.7)	<0.001
Age of diagnosis (y), mean (SE)	65.8 (0.2)	69.7 (0.6)	<0.001
Disease duration, mo	15.6	15.6	0.935
Sex, male, n (%)	1137 (64.5)	121 (72.5)	0.041
Ethnicity, White, n (%)	1701 (98.0)	164 (98.8)	0.766
BMI, mean (SE)	26.7 (0.1)	29.7 (0.4)	<0.001
Vascular risk category: number of vascular diseases (eg, angina, heart failure, stroke, heart attack, diabetes, high cholesterol, high blood pressure), n (%)	0 = 888 (51) 1 = 440 (25) >2 = 438 (25)	0 = 0 (0) 1 = 45 (27) >2 = 122 (73)	
Aspect of PD Scale			
Nonmotor symptoms			
UPDRS Part I, mean (SE)	9.2 (1.3)	10.2 (0.4)	0.004
NMSS total, mean (SE)	31.8 (0.7)	38.4 (2.5)	<0.001
Sleep, mean (SE)	5.9 (0.1)	7.1 (0.5)	0.014
Cardiovascular, mean (SE)	0.9 (0.04)	0.9 (0.1)	0.537
Mood, mean (SE)	4.7 (0.2)	6.0 (0.7)	0.066
Perception/Hallucinations, mean (SE)	0.6 (0.5)	0.7 (0.2)	0.608
Attention/Memory, mean (SE)	3.6 (0.1)	5.1 (0.4)	0.001
Gastrointestinal tract, mean (SE)	2.7 (0.1)	2.8 (0.3)	0.623
Urinary, mean (SE)	6.4 (0.2)	6.8 (0.5)	0.464
Sexual, mean (SE)	2.3 (0.1)	3.1 (0.3)	0.100
Miscellaneous, mean (SE)	4.0 (0.1)	4.6 (0.4)	0.393
Leeds Anxiety Index, mean (SE)	4.2 (0.1)	5.0 (0.3)	0.072
Anxiety (LADS > 6), n (%)	392 (23.1)	47 (30.3)	0.048
Leeds Depression Index, mean (SE)	4.2 (0.1)	5.1 (0.3)	0.003
Depression (LADS > 6), n (%)	380 (22.4)	55 (34.6)	0.001
Sleep, mean (SE)			
PDSS, mean (SE)	110.3 (0.6)	103.4 (1.9)	0.001
ESS, mean (SE)	6.7 (0.1)	7.9 (0.4)	0.001
Cognition			
MoCA total, mean (SE)	25.0 (0.1)	23.6 (0.3)	<0.001
MCI (MoCA score 21–25 and UPDRS 1.1 score < 4), n (%)	624 (35.7)	55 (33.1)	0.553
Psychiatric features			
ICD (QUIP 1–4 > 1.0), mean (SE)	136 (8.5)	19 (8.4)	0.973
ICD‐RDs (QUIP 5–8 > 1.0), mean (SE)	249 (17.1)	12 (9.6)	0.029
Hallucinations, n (%)	177 (10.1)	23 (13.9)	0.133
Motor features			
UPDRS Part II, mean (SE)	9.7 (0.2)	10.3 (0.5)	0.221
UPDRS Part III, mean (SE)	22.5 (0.3)	25.8 (0.9)	0.002
Substantial gait impairment, n (%)	46 (2.6)	20 (12.1)	<0.001
UPDRS Part IV, mean (SE)	0.7 (0.1)	0.9 (0.1)	0.288
Dyskinesia, n (%)	69 (4.0)	7 (4.2)	0.890
Quality of life			
PDQ8 total, mean (SE)	5.8 (0.1)	6.4 (0.4)	0.157
EQ5D Visual Analogue Scale, mean (SE)	77.2 (0.4)	72.0 (1.4)	<0.001
EQ5D Index, mean (SE)	0.74 (0.1)	0.68 (0.02)	0.001
SE‐ADL, mean (SE)	88.5 (0.3)	85.2 (0.9)	<0.001
Loss of independence, n (%)	150 (8.6)	33 (20.1)	<0.001
Hoehn & Yahr > 3, n (%)	142 (8.0)	23 (17.7)	0.001
Medication			
Levodopa equivalent daily dose (mg), mean (SE)	289.1 (4.6)	321.6 (15.2)	0.042
Untreated, n (%)	179 (10.2)	5 (3.0)	0.004

^a^ Means are estimated/adjusted for covariates, and *P* values are corrected for multiple comparisons.

PD, Parkinson's disease; T2DM, type 2 diabetes mellitus; BMI, body mass index; UPDRS, Unified Parkinson's Disease Rating Scale; NMSS, Non‐Motor Symptoms Scale; LADS, Leeds Anxiety and Depression Scale; PDSS, Parkinson's Sleep Scale; ESS, Epworth Sleepiness Scale; MoCA, Montreal Cognitive Assessment; MCI, mild cognitive impairment; ICD, impulse control disorder; ICD‐RD, impulse control and related disorders; QUIP, Questionnaire for Impulsive‐Compulsive Disorders in PD; PDQ8, Parkinson's Disease Questionnaire; SE‐ADL, Schwab and England Activities of Daily Living Scale.

Compared with the PD group, patients with comorbid T2DM were 3.9 years older than the PD group (*P* < 0.001), were older when diagnosed (69.7 vs. 65.8 years; *P* < 0.001), and had a higher BMI (29.7 vs. 26.7; *P* < 0.001).

### Impact of T2DM in Patients Recently Diagnosed with PD

Despite a similar disease duration of 15.6 months, and after additionally adjusting for differences in age, sex, ethnicity, vascular score, baseline H&Y stage, LEDD, and BMI, patients with T2DM had consistently more severe symptomatology in most aspects of PD (Table 1), including significantly worse overall nonmotor symptoms, as assessed by total MDS‐UPDRS Part I (10.2 [SD 0.4] vs. 9.2 [SD 0.1]; *P* = 0.004) and NMSS (38.4 [2.5] vs. 31.8 [0.7]; *P* < 0.001); worse sleep scores, as assessed by Parkinson's Sleep Scale (103.4 [1.9] vs. 110.3 [0.6]; *P* = 0.001) and Epworth Sleepiness Scale (7.9 [0.4] vs. 6.7 [0.1]; *P* = 0.001); worse cognitive scores, as assessed by MoCA (23.6 [0.3] vs. 25.0 [0.1]; *P* < 0.001); more severe motor symptoms, as assessed by MDS‐UPDS Part III (25.8 [0.9] vs. 22.5 [0.3]; *P* = 0.002); worse overall quality‐of‐life scores, as assessed by the EQ5D Visual Analogue Scale (72.0 [1.4] vs. 77.2 [0.4]; *P* < 0.001) and EQ5D Index (72.0 [1.4] vs. 77.2 [0.2]; *P* = 0.001); and higher impaired scores on the Schwab and England Activities of Daily Living scale (85.2 [0.9] vs. 88.5 [0.3]; *P* < 0.001) compared with people without T2DM. Also, patients with T2DM had a higher LEDD usage than participants without T2DM (321.6 [15.2] vs. 289.1 [4.6] mg; *P* = 0.042).

There were significantly greater numbers of patients with T2DM who had substantial gait impairment (12.6% vs. 2.6%; *P* < 0.001), depression (34.6% vs. 22.4%; *P* = 0.001), and self‐reported loss of independence (20.1% vs. 8.6%; *P* < 0.001) in comparison with patients without T2DM (Table 1). Patients with T2DM also had fewer ICD‐RD behaviors than patients without T2DM (9.6% vs. 17.1%; *P* = 0.029).

**FIG 2:**
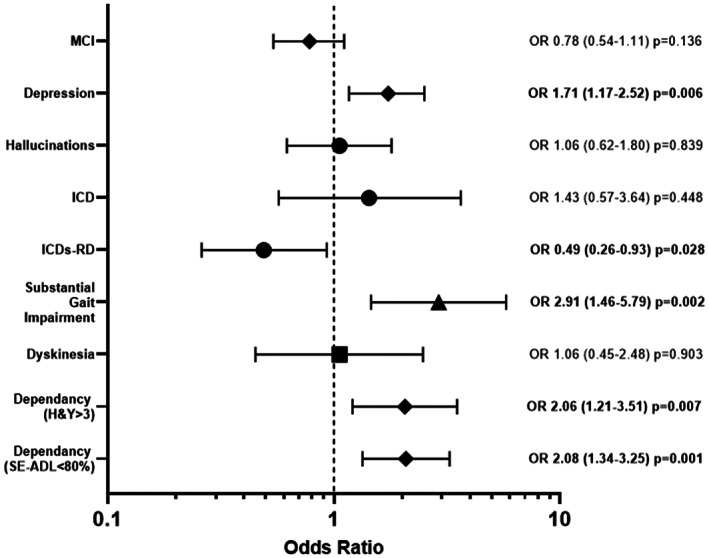
Likelihood of complications or reaching disease milestones in patients with Parkinson's disease (PD), according to type 2 diabetes mellitus (T2DM) status. Patients with T2DM were significantly more likely to have depression, substantial gait impairment, and loss of independence and were significantly less likely to have dopamine dysregulation than patients without T2DM. MCI, mild cognitive impairment; H&Y, Hoehn & Yahr; ICD, impulse control disorder; ICDs‐RD, impulse control and related disorders; OR, odds ratio; SE‐ADL, Schwab and England Activities of Daily Living Scale.

A multivariate binomial regression analysis showed that the T2DM was significantly and independently associated with greater gait impairment (OR, 2.91; 95% confidence interval [CI]: 1.46–5.79; *P* = 0.002), depression (OR, 1.62; CI: 1.10–2.39; *P* = 0.015), and loss of independence (OR, 2.08; CI: 1.34–3.25; *P* = 0.001) relative to the PD group, after adjusting for age, sex, disease duration, ethnicity, vascular score, LEDD, H&Y stage, and BMI (Fig. 2).

### Longitudinal Impact of T2DM on Development of Clinical Milestones

**FIG 3:**
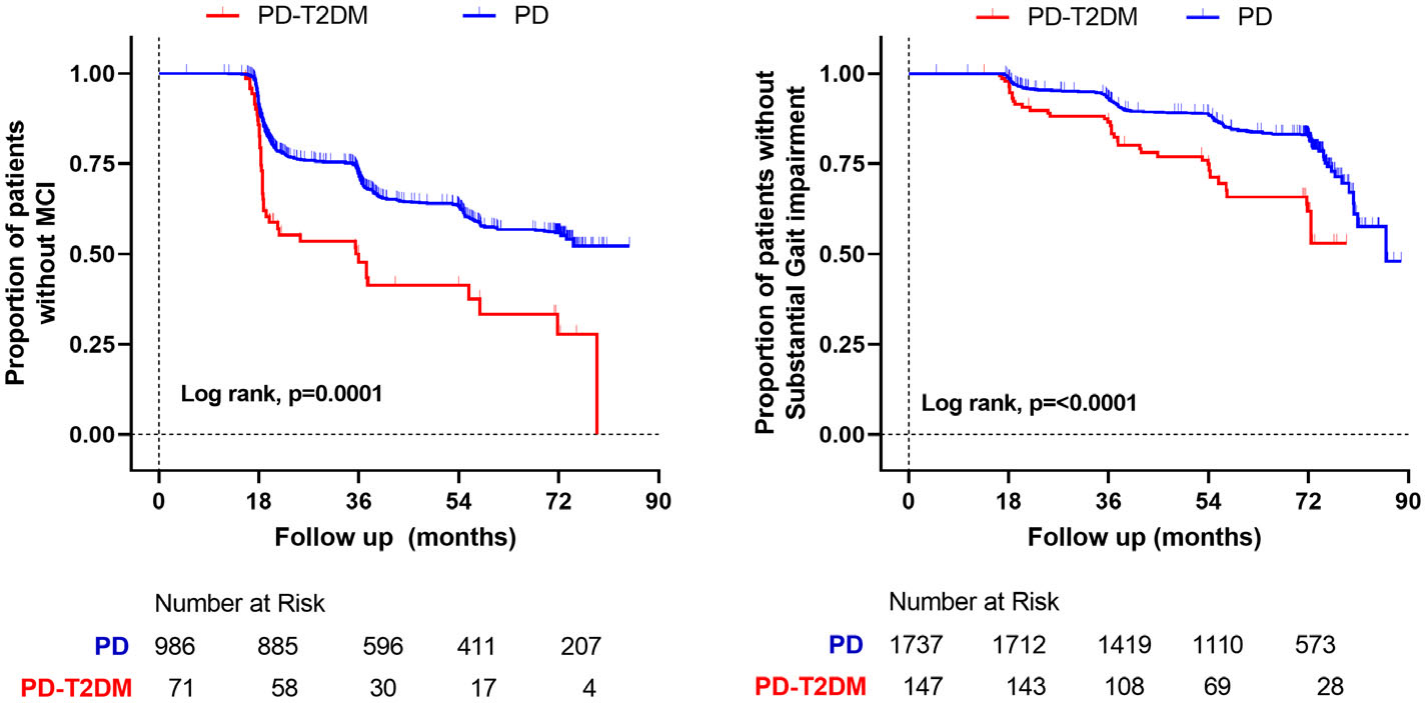
Timeline for the development of mild cognitive impairment (MCI) and substantial gait impairment, comparing Parkinson's disease (PD) cases with and without type 2 diabetes mellitus (T2DM). Kaplan–Meier curves show the significantly shorter time to develop both of these complications in patients with T2DM. [Color figure can be viewed at wileyonlinelibrary.com]

Over the total follow‐up period, MCI developed in 40 (56%) of 71 patients with PD + T2DM and 340 (34%) of 986 patients with PD without T2DM. Substantial gait impairment developed in 36 (24%) of 147 patients with PD + T2DM and in 232 (13%) of 1737 patients with PD (Fig. 3). Adjusting for differences in age, sex, PD duration, H&Y stage, vascular risk score, LEDD, and BMI, Cox proportional hazard survival analysis indicated T2DM was a predictor for patients to develop substantial gait impairment (hazard ratio, 1.55; CI: 1.07–2.23; *P* = 0.020) and also MCI (hazard ratio, 1.74; CI: 1.19–2.55; *P* = 0.004), compared with the PD (without T2DM) group. There were no significant differences in the time to develop H&Y stage 3, dyskinesia, motor fluctuations, hallucinations, ICD‐RDs, loss of independence, and depression (Supporting Information Fig. S2).

### Longitudinal Impact of T2DM on Progression of PD Symptoms

**FIG 4:**
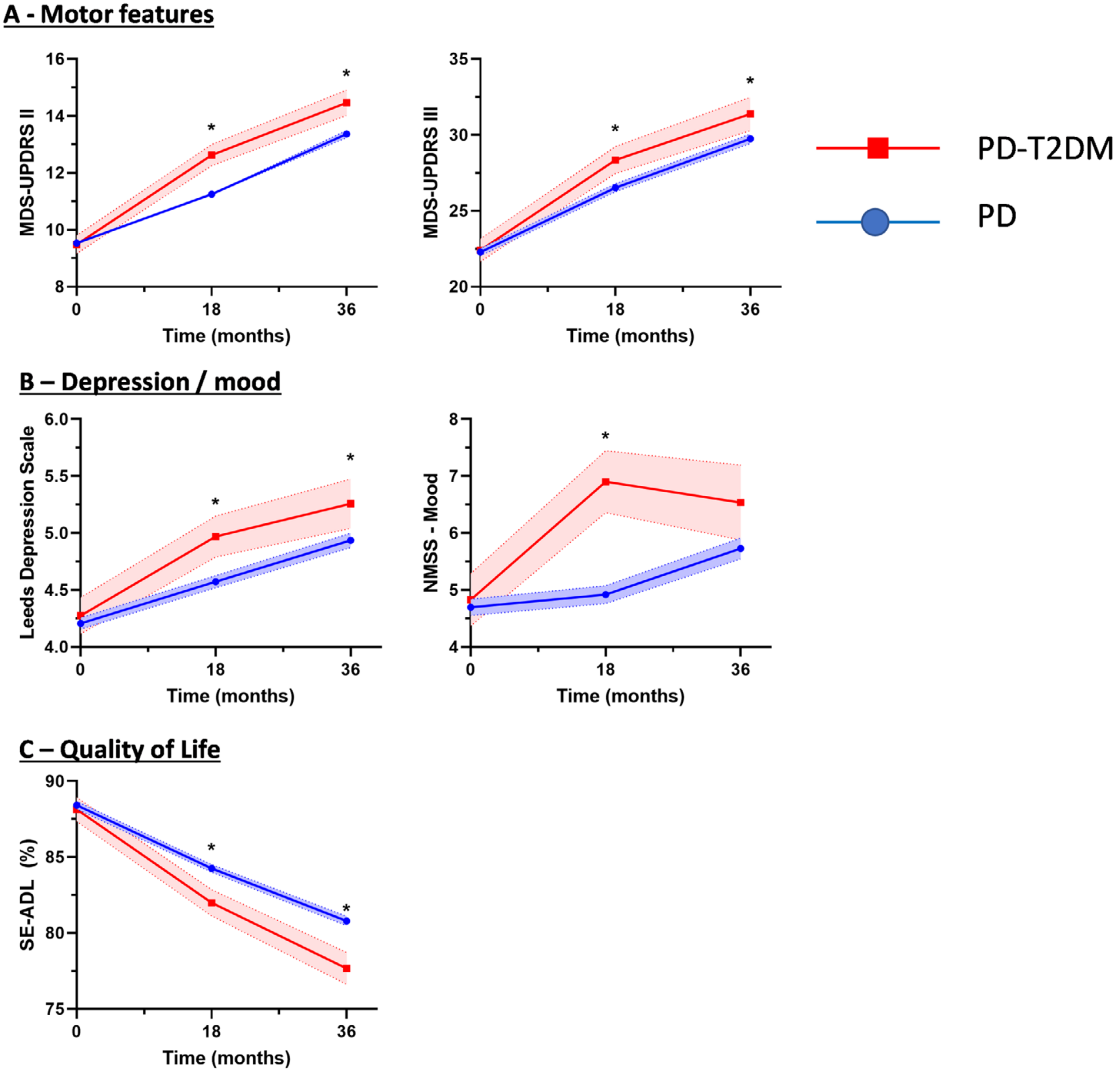
Time course of features in patients with Parkinson's disease (PD) comparing those with and without type 2 diabetes mellitus (T2DM). Progression was significantly faster for several domains. *P < 0.05. MDS‐UPDRS, Movement Disorder Society Unified Parkinson's Disease Rating Scale; NMSS, Non‐Motor Symptoms Scale; SE‐ADL, Schwab and England Activities of Daily Living Scale. [Color figure can be viewed at wileyonlinelibrary.com]

Patients were followed up for a mean of 36 months. When modeling the change in motor symptoms (MDS‐UPDRS Part II), there was a significant group × time interaction in the mixed model (*P* = 0.012), after adjusting for age, sex, gender, ethnicity, baseline motor score, LED, and BMI, indicating that there was a significant difference in the progression of motor symptoms in patients with T2DM. During the follow‐up, patients with T2DM had significantly more severe motor symptoms as reported by MDS‐UPDRS Part II scores. In addition, this group also had significantly worse MDS‐UPDRS Part III scores at each time point (Fig. 4).

There was also a significant group × time interaction when modeling change in mood as measured by the NMSS Mood subscore (*P* = 0.041), suggesting that the PD‐T2DM group had a significant difference in progression of mood symptoms. Supporting this, patients in the PD + T2DM group had significantly worse depression scores at each time point (*P* = 0.023).

Patients in the PD‐T2DM group also had significantly worse quality‐of‐life scores over the follow‐up period compared with the PD group (*P* = 0.001), with only the PD + T2DM group reporting loss of independence compared with the PD group (77.7% [1.0] vs. 80.8% [0.3]; *P* = 0.04) after 36 months; however, the group × time interaction failed to reach the conventional threshold for significance (*P* = 0.077). There were no significant differences in motor fluctuations or dyskinesia (Supporting Information Fig. S1).

### Exploratory Analysis of the Impact of Metformin in Patients with T2DM on Clinical Markers and Progression of PD

There were no consistent effects of metformin on PD symptoms in patients recently diagnosed with PD, and metformin did not offer any additional benefit in slowing the development of key clinical milestones (Supporting Information Table S3; Supporting Information Figs. S4, S5, and S6).

## Discussion

We evaluated the impact of T2DM on symptom severity and disease progression in patients recently diagnosed with PD. First, at baseline entry into the study, after a disease duration of 15 months, T2DM was independently associated with significantly more severe motor symptoms, greater total nonmotor symptoms, poorer cognitive scores as assessed by the MoCA, and being on greater amounts of dopaminergic medication. In addition, T2DM conferred an increased risk of patients having depression and substantial gait impairment. The PD + T2DM group had significantly worse quality‐of‐life scores and increased dependency than patients with PD without T2DM. Second, over time, patients with T2DM had significantly faster progression of motor symptoms, worse depression scores, and were more likely to develop substantial gait impairment and MCI than patients without T2DM. Overall, these findings suggest T2DM is an independent factor for more severe PD and also negatively alters long‐term outcomes.

Our findings that patients with PD with T2DM have more aggressive disease are in line with earlier smaller studies (Supporting Information Table S1). The question of whether the effects of T2DM on PD are simply additive or are interactive is uncertain. Although it is well‐known that people with multiple chronic conditions tend to have poorer outcomes,^29^ there is accumulating evidence that biological mechanisms and pathways involved in T2DM at the cellular level may trigger or interact with pathways involved in PD pathogenesis. For example, patients with T2DM who do not have PD show signs of subclinical striatal dopaminergic dysfunction on DaTscans.^11^ Similarly, healthy mice fed a high‐fat diet to induce peripheral insulin resistance demonstrate nigrostriatal dopaminergic dysfunction and parkinsonism,^30^ supporting pathophysiological associations between PD and T2DM. This supports the notion that T2DM and PD are likely synergistic conditions linked by dysregulated pathophysiological pathways, rather than two coincidental aging processes.

One potential explanation for the effects of T2DM on PD phenotype may be as a result of increased neurovascular burden. Leukoaraiosis is more common in T2DM; in PD, it has been associated with the severity of motor impairment.^31^ However, this link is largely not supported by epidemiological studies, in which the association of T2DM to PD persists after the exclusion of patients with vascular disease,^3^, ^32^ and imaging studies have shown similar levels of leukoaraiosis between PD and PD + T2DM groups despite worse gait and cognitive impairment in the latter.^13^, ^33^ Furthermore, our sensitivity analysis in this study (Supporting Information Tables S4 and S5; Supporting Information Fig. S7) suggests mechanisms other than increased cerebrovascular disease are contributing to increased disease severity.

It is generally accepted that abnormal spreading of pathological α‐synuclein, in a prion‐like manner, causes disease propagation in PD; similarly, aberrant folding of islet amyloid polypeptide (IAPP) protein in pancreatic beta islets is thought to contribute to the development of T2DM.^34^ In addition, there appears to be the potential for interaction between these two proteins, which may trigger and exacerbate pathology in these diseases. IAPP and α‐synuclein, as well as Aβ and tau, have been found to be colocalized in pancreatic β cells in patients with synucleinopathies, which may underlie the appearance of insulin resistance in non‐T2DM AD, PD, or dementia with Lewy bodies patients.^35^, ^36^ Furthermore IAPP can interact with and accelerate α‐synuclein aggregation in vitro (although the converse is not true), providing a simple theoretical justification for why T2DM is a risk factor for PD, whereas patients with PD do not have an increased risk of developing T2DM.^37^ Supporting this are recent studies demonstrating significantly elevated amylin and pathogenic α‐synuclein in the substantia nigra in patients with PD compared with healthy control subjects.^36^, ^38^ Thus, the aberrant heterologous cross seeding of these proteins remains a further intriguing theory that suggests patients with PD with T2DM may represent a more severe subtype of “body‐first” PD, where the pathology originates in the enteric or peripheral autonomic nervous system and then ascends via the vagus nerve and sympathetic connectome to the CNS,^39^, ^40^ and thus may represent a window of opportunity to treat and possibly eventually prevent PD.

This study is the first to report the impact of T2DM on nonmotor symptoms in PD. At study entry, in patients with a mean disease duration of 18 months, patients with T2DM already had a greater overall nonmotor symptom burden (as measured by MDS‐UPDRS Part I and NMSS) and reported poorer sleep compared with patients without T2DM. Interestingly, the main drivers for the differences in total NMSS scores were primarily sleep, mood, and memory issues, which were themselves captured on separate scales.

Previous studies suggested patients with PD with T2DM were more likely to have cognitive impairment after a mean of 6 years' disease duration; however, in this study, we show even 18 months after a diagnosis of PD, comorbid T2DM was associated with significantly worse cognitive test scores and a higher proportion of MCI. In addition, in patients without cognitive impairment at baseline, the PD + T2DM group was almost twice as likely to develop MCI subsequently. Although increased leukoaraiosis in these patients has previously been thought to underlie these effects, our study did not necessitate structural imaging, and we were not able to perform a systematic analysis of this topic. However, others demonstrate no difference in leukoaraiosis between patients with PD with and without T2DM.^13^, ^41^ An alternative mechanism is via decreased insulin signaling. Insulin resistance is associated with cognitive dysfunction^42^ and, even in de novo PD patients in the prediabetic range, was shown to lead to faster rates of decline in cognitive performance than nondiabetic/normoglycemic patients with PD.^15^ Dysfunctional neuronal insulin signaling leads to increased aggregation of amyloid‐ß, hyperphosphorylated τ, proinflammatory pathway activation, and impaired brain glucose metabolism,^43^ and thus may explain accelerated decline in cognition in PD. Furthermore, it has long been established that molecular interactions between pathological proteins may occur within the same brain in various distribution patterns, causing variable phenotypes and mixed pathologies, and so it is possible that T2DM, via dysregulated insulin signaling, may lead to promotion of AD pathology in a subset of patients with PD, increasing the risk of developing cognitive impairment.

The PD + T2DM group had significantly worse mood scores at baseline and reported consistently lower mood throughout follow‐up than the PD group. Furthermore, T2DM was independently and directly associated with worse longitudinal progression of the NMSS mood subscores throughout the follow‐up period. Depression is a strong predictor of quality of life in PD, and is itself a risk factor for PD, while similarly, there is increased prevalence of depression in patients with T2DM.^44^ Insulin resistance has been identified as a risk factor for depression,^45^, ^46^, ^47^ and even nondiabetic patients with depressive disorders exhibit elevated levels of brain insulin resistance markers^48^, ^49^; thus, insulin resistance may be a shared pathological mechanism that may trigger or exacerbate depression in PD. The mechanisms linking insulin resistance, depression, and PD are uncertain but may involve insulin signaling–modulated activation of proinflammatory pathways, exacerbation of defective synaptic plasticity, and impairment of the normal hypothalamic–pituitary–adrenal axis, leading to the dysfunction of physiological mechanisms of reward.^50^ Despite this uncertainty, the identification of T2DM as a risk factor for depression in PD is important^51^ and may aid clinicians in identifying patients at increased risk of depressive disturbances.

Contrary to other nonmotor symptoms, patients with T2DM reported fewer symptoms of ICD‐RDs. The pathophysiology of ICD‐RDs like punding is complex but is thought to involve stimulation of D1 and D2 receptors, and studies in animals support the hypothesis that the reward system acts by means of increasing dopamine in the nucleus accumbens and the dorsal striatum (becoming conditioned cues).^52^ Interestingly, insulin signaling has a reciprocal relationship to dopamine action and impacts behaviors such as reward and mood, and clinical studies have shown insulin resistance is associated with less endogenous dopamine at D2/3 receptors^53^; thus, patients with T2DM may be at lower risk for these behaviors.

Given the interest in antiglycemic drugs as potential novel treatments for PD, an exploratory analysis was performed to evaluate whether metformin could reduce or restore the negative impact of T2DM. This study demonstrated that metformin use did not confer any protective effects on the diabetic PD population on any outcomes, and in fact, MoCA scores were significantly lower (worse) at 36 months in the PD + T2DM/metformin group. Interest in metformin as a potential neuroprotective drug is supported by in vivo and in vitro studies demonstrating metformin can restore dopaminergic dysfunction and reduce aggregation of α‐synuclein.^54^ However, results from other studies are conflicting. Studies have shown metformin increases the risk of developing dementia and PD^55^, ^56^ and can exacerbate intracellular and extracellular production of amyloid‐ß.^57^ Collectively, our data may imply that metformin does not offer additional benefit on neurological outcomes in diabetes, but a more robust, appropriately designed study is needed to better inform on this issue.

The main strength of our study is that this large longitudinal study of patients with PD was deeply phenotyped with a variety of PD scales to characterize a variety of symptoms, in addition to typical motor scales, allowing us to gain a global overview of PD severity, as well as individual symptoms. We also had a large number of relevant vascular risk factor covariate data available. Using these and other data in our directed acyclic graph allowed us to select many covariates (based on a literature review and expert opinion) that allowed us to adjust for any potential differences between groups in our models to help estimate causal effects from the observed data. This was helpful in delineating and understanding confounders and potential sources of bias, and examining the independent effects of T2DM in sequential analyses. The prevalence of T2DM in our PD cohort was approximately 10%, which is in line with other reported studies, and thus the large number of patients with T2DM allowed tentative casual inferences to be made and some generalizability of the findings. Interestingly, at baseline, the PD + T2DM group was significantly older and had later disease onset compared with the PD group. Previous studies have shown the greatest risk of developing PD occurs in those T2DM patients with longer disease duration, and synergistic effects of T2DM on PD tend to occur at later stages, possibly explaining the later age of onset.^3^, ^10^ However, both age and age of onset of PD have been shown to impact disease progression and so these differences may have impacted subsequent analysis, despite our best efforts to take this into account.

An important limitation in this study, as is observed in many other longitudinal studies of this nature, is the dropout of patients during follow‐up. Dropout was significant in both groups even by month 32, but higher in the PD + T2DM group, which suggests that data may not be missing at random. One could speculate that this is driven by higher disease severity, which would support our overall findings of the negative impact of T2DM on PD severity. However, because this may introduce a bias regarding surviving patients, a decision was made to include follow‐up data for only the first 36 months for one aspect of the longitudinal analysis. The survival analyses curves may have been influenced by censored data, and further studies will be needed to confirm these findings.

We used linear mixed effects models to examine the interaction effects of T2DM on a number of clinical features typically associated with PD, and our results suggested worse motor progression and mood over time were dependent on the presence of T2DM. Thus, multivariable models can simultaneously control for many covariates and also be used to test additive versus interactive effects of different covariates, which can help generate new hypotheses about the effects of T2DM on PD. However, when there are potentially many covariates, a large sample size is required, although a variety of statistical approaches are available to address this.^58^ Although this is the largest study to date to evaluate the impact of T2DM on PD, the number of cases of PD + T2DM is still relatively small, may have impacted our analyses, and must be validated in further studies. T2DM was identified using medication data and self‐report, suggesting diabetes may have been underrecognized in this study. However, this typically would have weakened our ability to detect an association between PD and T2DM. In addition, this study was not able to fully examine different facets of T2DM phenotypes, such as the effects of diabetes severity, duration of disease, and presence of other diabetic comorbidities that may have impacted the relationship of T2DM to PD severity. Particularly challenging is controlling for changes to other non‐PD and diabetic medication made throughout the follow‐up. Further evaluation of cohorts using well‐phenotyped diabetic populations would be needed to fully understand these interactions. Although we had extensive data on patients' vascular risk factors available in this cohort, and we attempted to account for these in our statistical models and sensitivity analysis, we note that using an aggregate vascular score as an ordinal covariate carries the risk of overweighing some elements of aggregate vascular comorbidity burden, while underweighting others.

In conclusion, we have identified T2DM as an independent risk factor associated with more severe motor symptoms, nonmotor symptoms, and poorer quality‐of‐life scores in patients recently diagnosed with PD. Furthermore, the presence of T2DM has a detrimental effect on the clinical course of PD, contributes to faster motor and nonmotor symptom progression, and increases the risk of developing MCI and gait impairment. The importance of this is that insulin resistance is potentially a modifiable metabolic state, with multiple peripheral and central targets for intervention. Thus, targeting insulin resistance may represent a novel target for alleviating parkinsonian symptoms, ameliorating neurodegeneration, and slowing progression to disability and dementia.^59^ Multiple trials of antiglycemic medications for the treatment of PD and other neurodegenerative diseases are currently underway, and the results of these will greatly inform the next generation of novel PD treatments.

## Author Roles

D.A.: conception, data analysis, and manuscript writing and editing. J.E., A.W., G.V., M.L.C., N.V., C.G., T.F., and S.G.: manuscript writing and editing. M.L.: data analysis and manuscript writing and editing. Y.B.‐S., H.M., and D.G.: study design, data collection, and manuscript editing.

## Financial Disclosures

D.A. has received honoraria from Bial Pharma and grant funding from CureParkinson's, Parkinson's UK, and National Institute for Health Research, outside of the submitted work. N.V. has received unconditional educational grants from IPSEN and Biogen, travel grants from IPSEN and AbbVie, and honorarium from AbbVie and STADA, and has served on advisory boards for AbbVie and Brittania, outside of the submitted work. Y.B.‐S. has received grant funding from the Medical Research Council, National Institute for Health Research, Parkinson's UK, National Institutes of Health, and Economic and Social Research Council. H.M. reports paid consultancy from Biogen, UCB, AbbVie, Denali, Biohaven, and Lundbeck; lecture fees/honoraria from Biogen, UCB, C4X Discovery, GE‐Healthcare, Wellcome Trust, and Movement Disorders Society; research grants from Parkinson's UK, Cure Parkinson's Trust, PSP Association, CBD Solutions, Drake Foundation, and Medical Research Council. D.G. received honoraria from Bial Pharma, Merz Pharma, and consultancy fees from The GM Clinic, Glasgow. T.F. has received honoraria from Profile Pharma, BIAL, AbbVie, Genus, Medtronic, and St. Jude Medical, outside of the submitted work. All other authors have nothing to report.

## Supporting information


**Table S1.** Previous studies evaluating the effects of T2DM on PD *disease progression*.
**Table S2.** Longitudinal follow up. Univariate and multivariate Cox regression analyses for the development of each disease marker/clinical outcome in PD according to the presence of T2DM. Multivariate analyses adjusted for age, sex, vascular score, ethnicity, disease duration, Hoehn & Yahr stage, BMI and LEDD.
**Table S3.** Clinical Features of patients within 18 months of diagnosis of PD compared to patients with co‐morbid T2DM and patients treated with metformin.
**Table S3.** Multivariate Cox regression analyses for the development of each disease marker/clinical outcome in patients with PD according to the use of metformin. Multivariate analyses adjusted for age, sex, vascular score, ethnicity, disease duration, Hoehn & Yahr stage, BMI and LED.
**Table S4.** Clinical Features of patients within 18 months of diagnosis of PD. Patients were categorised into patients with no T2DM and no vascular risk factors (PD/VasR−), patient with no T2DM but increased vascular risk factors (PD/VasR+), and patients with co‐morbid T2DM (PD + T2DM). There were no significant differences between the PD/VasR− and PD/VasR+ groups, but when compared to the increased vascular risk factors group (without T2DM), patients with T2DM had significantly worse scores in depression, quality of life and substantial gait impairment.
**Table S5.** Multivariate Cox regression analyses for the development of each disease marker/clinical outcome in patients with PD according to the use of metformin. Multivariate analyses adjusted for age, sex, ethnicity, disease duration, Hoehn & Yahr stage, BMI and LED.
**Figure S1.** Directed acyclic graph illustrating confounding and mediating factors to determine the causal impact of T2DM on PD severity. This considers each variable in relation to the exposure and outcome, as both the failure to adjust for a confounder, and over‐adjusting for an intermediate variable can lead to biased results^16,17^. Included associations were based on past literature and expert knowledge, and we used the program DAGitty^18^, which uses an algorithm to identify a “minimally sufficient adjustment set” containing no redundant variables, to allow us to adjust models for confounders and make causal inferences.
**Figure S2.** Kaplan–Meier curves – no significant differences were observed in time to develop H&Y Stage 3, Dyskinesia, Motor fluctuations, Hallucinations, ICD, Loss of independence, Depression.
**Figure S3.** Linear mixed modelling progression of symptoms of PD.
**Figure S4.** Longitudinal impact of T2DM on symptoms in PD per group. Multivariate Cox regression analyses for the development of each disease marker/clinical outcome in PD according to the presence of T2DM. Multivariate analyses adjusted for age, sex, ethnicity, disease duration, vascular score, Hoehn & Yahr stage, BMI and LEDD.
**Figure S5.** Longitudinal impact of T2DM on symptoms in PD per group.
**Figure S6.** Linear mixed modelling progression of symptoms of PD vs PD + T2DM and PD + T2DM/Met.
**Figure S7.** Longitudinal follow up. Univariate regression analyses for the development of each disease marker/clinical outcome in PD according to the presence of no vascular risk factors (PD/VasR−), increased vascular risk factors (PD/VasR+), and T2DM.

## Data Availability

The data that support the findings of this study are available on request from the the Tracking‐PD study
