# Supplementary material for: The Impact of Type 2 Diabetes in Parkinson's Disease
Source: Mov Disord. 2022 Jun 14;37(8):1612–23. doi: 10.1002/mds.29122 (PMC9543753; doi:10.1002/mds.29122)
Supplement: Supplementary file 1 — Table S1. Previous studies evaluating the effects of T2DM on PD disease progression. Table S2. Longitudinal follow up. Univariate and multivariate Cox regression analyses for the development of each disease marker/clinical outcome in PD according to the presence of T2DM. Multivariate analyses adjusted for age, sex, vascular score, ethnicity, disease duration, Hoehn & Yahr stage, BMI and LEDD. Table S3. Clinical Features of patients within 18 months of diagnosis of PD compared to patients with co‐morbid T2DM and patients treated with metformin. Table S3. Multivariate Cox regression analyses for the development of each disease marker/clinical outcome in patients with PD according to the use of metformin. Multivariate analyses adjusted for age, sex, vascular score, ethnicity, disease duration, Hoehn & Yahr stage, BMI and LED. Table S4. Clinical Features of patients within 18 months of diagnosis of PD. Patients were categorised into patients with no T2DM and no vascular risk factors (PD/VasR−), patient with no T2DM but increased vascular risk factors (PD/VasR+), and patients with co‐morbid T2DM (PD + T2DM). There were no significant differences between the PD/VasR− and PD/VasR+ groups, but when compared to the increased vascular risk factors group (without T2DM), patients with T2DM had significantly worse scores in depression, quality of life and substantial gait impairment. Table S5. Multivariate Cox regression analyses for the development of each disease marker/clinical outcome in patients with PD according to the use of metformin. Multivariate analyses adjusted for age, sex, ethnicity, disease duration, Hoehn & Yahr stage, BMI and LED. Figure S1. Directed acyclic graph illustrating confounding and mediating factors to determine the causal impact of T2DM on PD severity. This considers each variable in relation to the exposure and outcome, as both the failure to adjust for a confounder, and over‐adjusting for an intermediate variable can lead to biased results16,17. In [file MDS-37-1612-s001.docx]

**Supplementary Material**

Table 1: Previous studies evaluating the effects of T2DM on PD *disease progression*

| Author and  Study design | Sample size | Outcome(s) | Findings between PD and PD+DM groups |
| --- | --- | --- | --- |
| Pablo-Fernandez et al, 2021  Cohort | N=135  107 PD, 25 PD+T2DM | Time to disability milestones | PD+T2DM developed earlier falls, wheelchair dependence, dementia, care home admission and reduced survival |
| Ou et al, 2021^1^  Prospective | N=379;  330 PD, 49 PD+T2DM  *(49 PD+T2DM further divided into poorly controlled T2DM (n=22), well-controlled, n=27)* | Survival;  Motor decline (>14pt decline in UPDRS III and/or >H&Y Stage 3);  Cognitive decline (>3pt decrease on MoCA). | No change in survival or cognitive decline  Poorly controlled DM associated with faster time to reach H&Y Stage 3 (HR 2.1 (95%CI 1.2‐3.5) compared to well controlled T2DM |
| Chung et al, 2019^1^  Case series | N=549  463 PD, 86 PD+T2DM | Change in LEDD | T2DM associated with greater increase in LEDD than those without T2DM. |
| Pagano et al, 2018^2^  Prospective | N=78;  25 PD, 25 PD+T2DM, 14 T2DM, 14 HC | Motor decline (>1pt change in H&Y stage)  Cognitive decline (self- report and neuropsychiatric tests) | T2DM associated with faster motor decline (HR 4.5, 95%CI 1.4–13.9) and cognitive decline (HR 9.3, 95%CI 1.1–74.5) |
| Mollenhauer et al, 2018^3^  Observational | N= 244  122 PD, 13 PD+T2DM, 109 HC | Annual change in motor decline (UPDRS III scores) and cognitive decline (MMSE) | T2DM associated with cognitive decline |
| Ibrahim et al, 2018^4^  Cross-sectional | N=72  51 PD, 21 PD+T2DM  *(6 of the 21 PD+T2DM group developed T2DM after PD diagnosis, but were included in analysis)* | Onset to motor complications | PD+T2DM developed motor complications 12 months before PD group |

**Supplementary Figure 1:**

Directed acyclic graph illustrating confounding and mediating factors to determine the causal impact of T2DM on PD severity. This considers each variable in relation to the exposure and outcome, as both the failure to adjust for a confounder, and over-adjusting for an intermediate variable can lead to biased results^16,17^. Included associations were based on past literature and expert knowledge, and we used the program DAGitty^18^, which uses an algorithm to identify a “minimally sufficient adjustment set” containing no redundant variables, to allow us to adjust models for confounders and make causal inferences.


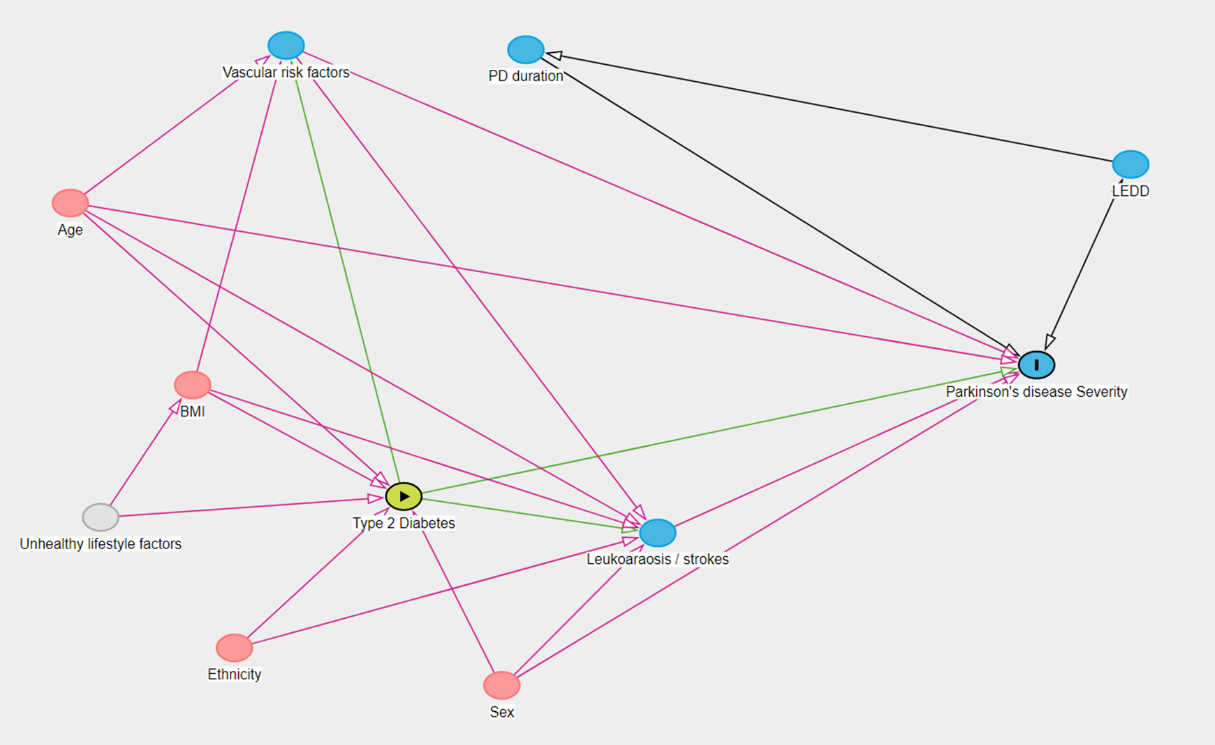

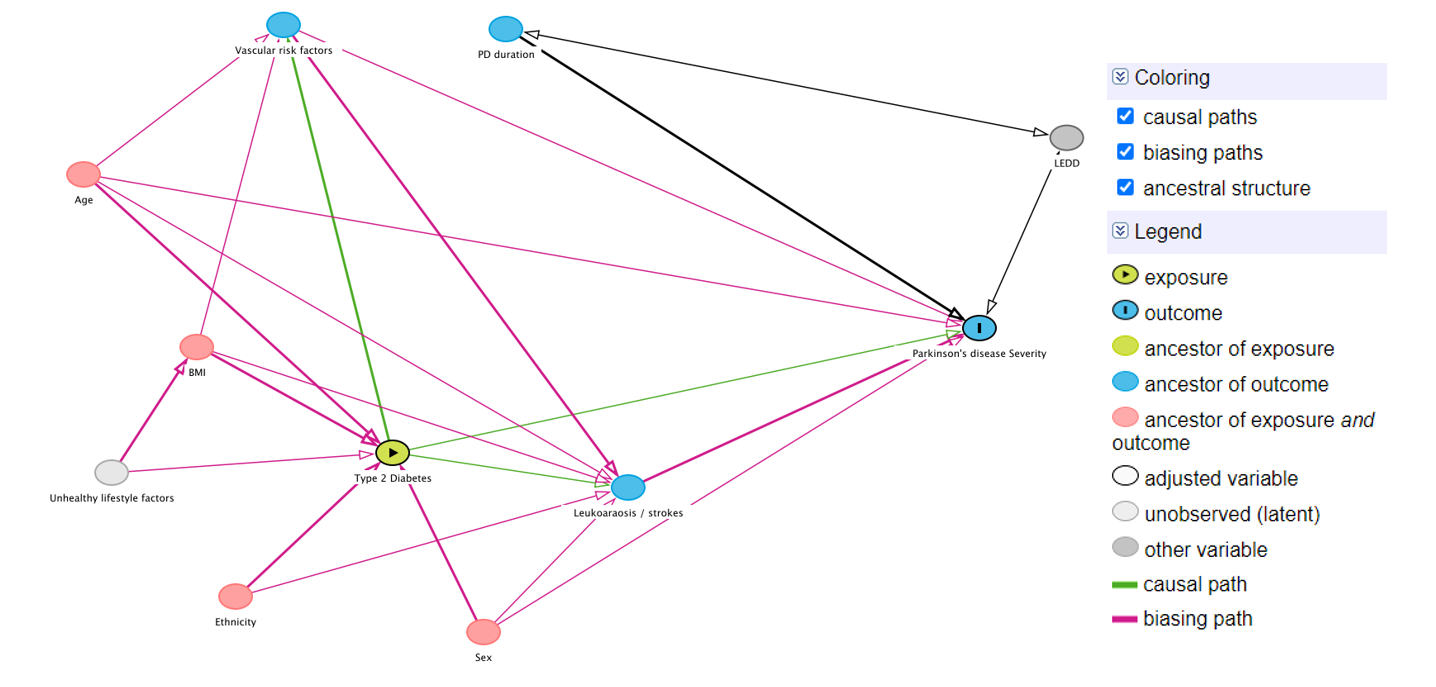


**Supplementary Table 2:**

Longitudinal follow up. Univariate and multivariate Cox regression analyses for the development of each disease marker / clinical outcome in PD according to the presence of T2DM. Multivariate analyses adjusted for age, sex, vascular score, ethnicity, disease duration, Hoehn & Yahr stage, BMI and LEDD.

| Disease marker / clinical milestone | Univariate  HR (95% CI) | p value | Multivariate  HR (95% CI) | p value |
| --- | --- | --- | --- | --- |
| **Substantial gait impairment**  *(MDS-UPDRS 3·10>3)* | 2·33 (1·64-3·32) | <0·0001 | 1·55 (1·07-2·23) | 0·020 |
| **MCI**  *(MoCA<26)* | 2·16 (1·56-3·01) | <0·0001 | 1·74 (1·19-2·55) | 0·004 |
| **Hallucinations**  *(MDS-UPDRS 1·2>1)* | 0·91 (0·68-1·21) | 0·503 | 0·80 (0·59-1·08) | 0·139 |
| **Depression**  *(LADS>6)* | 1·42 (1·0-2·02) | 0·051 | 1·32 (0·92-1·90) | 0·134 |
| **ICD**  (QUIP 1-4 >1) | 1·13 (0·79-1·61) | 0·494 | 1·13 (0·78-1·65) | 0·524 |
| **Dyskinesia**  *(MDS-UPDRS 4·1>1)* | 1·12 (0·73-1·71) | 0·608 | 1·08 (0·67-1·73) | 0·748 |
| **Motor fluctuations**  *(MDS-UPDRS 4·3>1)* | 1·23 (0·91-1·65) | 0·185 | 1·25 (0·91-1·72) | 0·167 |
| **Loss of independence**  *(SE-ADL<80%)* | 1·56 (1·15-2·10) | 0·004 | 1·18 (0·89-1·61) | 0·289 |
| **Loss of independence**  (H&Y >3) | 1·56 (1·13-2·15) | 0·007 | 1·11 (0·79-1·56) | 0·548 |

MCI (Mild cognitive impairment); ICD (impulse control disorder); MDS-UPDRS (Movement Disorders Society Parkinson’s Disease Rating scale); H&Y (Hoehn & Yahr stage)

**Supplementary Figure 2:**


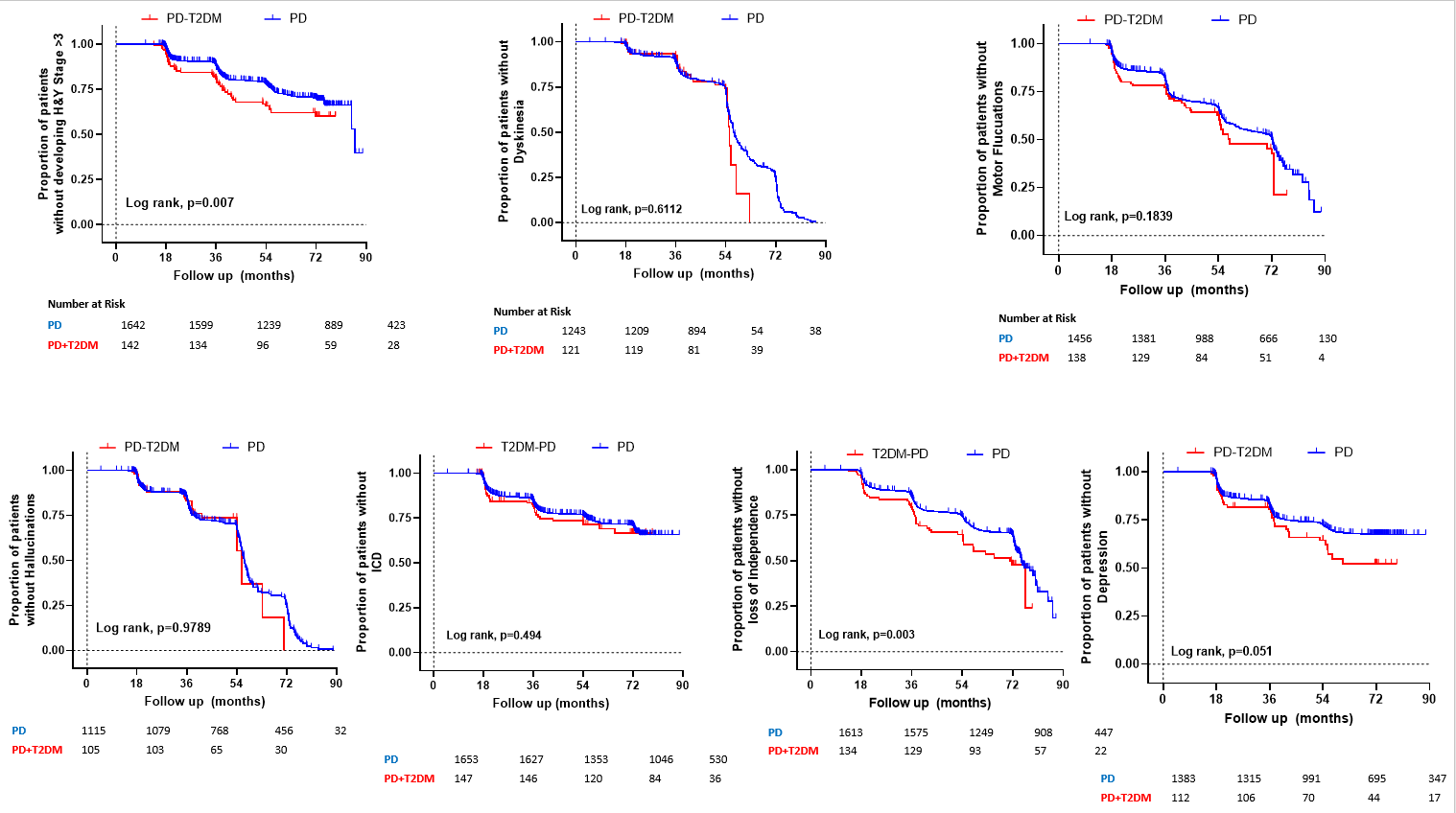
Kaplan Meier curves - no significant differences were observed in time to develop H&Y Stage 3, Dyskinesia, Motor fluctuations, Hallucinations, ICD, Loss of independence, Depression

MCI (Mild cognitive impairment); ICD (impulse control disorder); MDS-UPDRS (Movement Disorders Society Parkinson’s Disease Rating scale); H&Y (Hoehn & Yahr stage)

**Supplementary Figure 3:**

Linear mixed modelling progression of symptoms of PD


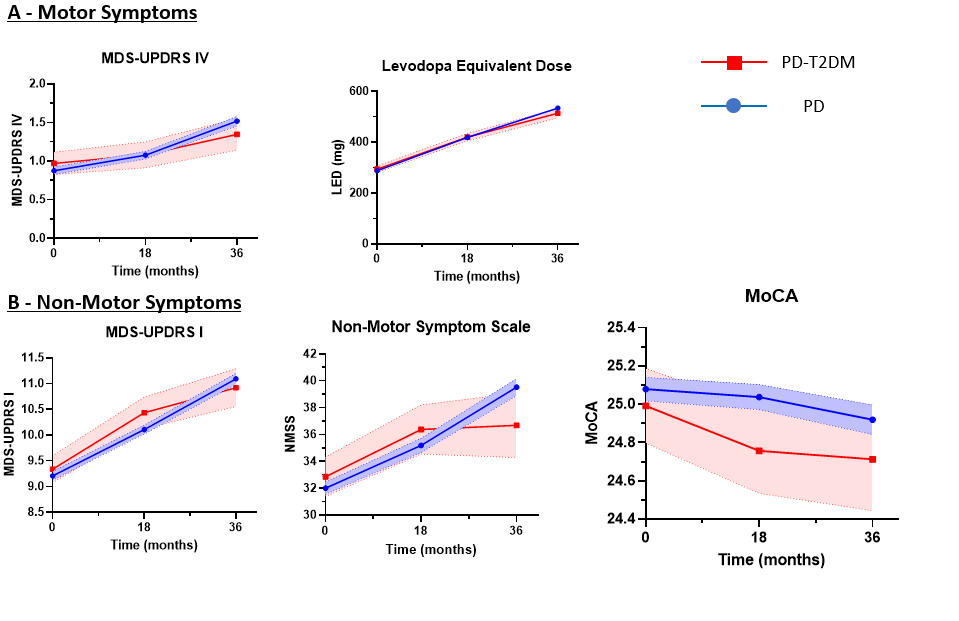


**Supplementary Table 3:**

Clinical Features of patients within 18 months of diagnosis of PD compared to patients with co-morbid T2DM and patients treated with metformin

| **Demographics** |  | | | **PD**  **(n=1763)**  **Mean (SE)*** | **PD-T2DM**  **(n=72)**  **Mean (SE)*** | **PD-T2DM/Met**  **(n=95)**  **Mean (SE)*** | **P value** |  |
| --- | --- | --- | --- | --- | --- | --- | --- | --- |
|  | Age | | | 67·19 (0·2) | 72·26 (0·9) | 70·11 (0·9) | <0.001 |  |
|  | Age of diagnosis | | | 65·85 (0·2) | 70·85 (0·9) | 68·84 (0·9) | <0.001 |  |
|  | Disease duration, years | | | 1·33 (0·1) | 1·41 (0·1) | 1·27 (0·1) | 0.590 |  |
|  | Sex, males (%) | | | 1137 (90·4) | 51 (69·9) | 70 (74·5) | 0·098 |  |
|  | Ethnicity, white (%) | | | 35 (2·0) | 1 (1·4) | 1 (1·1) | 0·761 |  |
|  | BMI | | | 26·74 (0·1) | 29·68 (0·6) | 29·67 (0·63) | <0.001 |  |
|  |  | | |  |  |  |  |  |
| **Aspect of PD** | **Scale** | | |  |  |  |  |  |
| **Non-motor symptoms** | **UPDRS I** | | | **9·18 (0·1)** | **11·1 (0·6)** | **9·98 (0·5)** | **0·006^a,b^** |  |
|  | **NMSS Total** | | | **31·69 (0·7)** | **39·4 (3·5)** | **37·28 (3·1)** | **0·030** |  |
|  | **Leeds Anxiety Index** | | | **4·10 (0·8)** | **4·52 (0·4)** | **4·7 (0·3)** | **0·149** |  |
|  |  | *Anxiety (LAD>6), n (%)* | | *392 (23·1)* | *18 (26·5)* | *29 (33·3)* | *0·078* |  |
|  | **Leeds Depression Index** | | | **4·22 (0·7)** | **5·06 (0·3)** | **5·03 (0·3)** | **0·009** |  |
|  |  | *Depression (LAD>6, n (%)* | | *380 (22·4)* | *22 (31·4)* | *33 (37·1)* | *0·002* |  |
|  |  | |  | | | | | |
| **Sleep** | **PDSS** | | |  |  |  |  |  |
|  | **ESS** | | | 6·71 (0·10) | 8·15 (0·5) | 7·56 (0·5) | 0·010^a^ |  |
|  |  | | |  |  |  |  |  |
| **Cognition** | **MOCA Total** | | | 25·08 (0·1) | 23·97 (0·4) | 23·33 (0·3) | <0·001^a,c^ |  |
|  |  | *MCI (MOCA<26), n (%)* | | 788 (48·2) | 39 (60·0) | *57 (67·1)* | 0·001 |  |
|  |  | |  | | | | | |
| **Psychiatric co-morbidity** |  | *Dopamine dysregulation* | | *37 (2·1)* | *4 (5·6)* | *2 (2·1)* | *0·156* |  |
|  |  | *ICD (QUIP>1·0)* | | *43 (2·6)* | *3 (4·3)* | *4 (4·7)* | *0·360* |  |
|  |  | *Hallucinations* | | *43 (2·5)* | *5 (6·9)* | *3 (3·2)* | *0·065* |  |
|  |  | |  | | | | | |
| **Motor symptoms** | **UPDRS II** | | | 9·71 (0·1) | 11·01 (0·7) | 9·84 (0·6) | 0·246 |  |
|  | **UPDRS III** | | | 22·50 (0·3) | 26·6 (1·5) | 25·1 (1·2) | 0·006^a^ |  |
|  |  | *Substantial gait impairment, n (%)* | | *46 (2·6)* | *9 (12·7)* | *11 (16·7)* | *<0·001* |  |
|  | **UPDRS IV** | | | 0·73 (0·1) | 0·8 (0·2) | 0·9 (0·2) | 0·625 |  |
|  |  | *Dyskinesia, n (%)* | | 69 (4·0) | 2 (2·8) | *5 (5·4)* | 0·696 |  |
|  |  | |  | | | | | |
| **Quality of life** | **PDQ8 Total** | | | 5·77 (0·1) | 6·78 (0·5) | 6·0 (0·4) | 0·213 |  |
|  | **EQ5D VAS** | | | 77·2 (0·4) | 70·1 (2·0) | 73·0 (1·7) | <0·001^a^ |  |
|  | **EQ5D Index** | | | 0·72 (0·1) | 0·66 (0·2) | 0·68 (0·1) | 0·004^a^ |  |
|  | **SE-ADL** | | | 88·5 (0·2) | 83·4 (1·3) | 86·4 (1·1) | <0·001^a^ |  |
|  |  | *Loss of independence, n (%)* | | 150 (8·6) | 19 (26·8) | 14 (15·1) | <0·001 |  |
|  |  | *Hoehn & Yahr >3, n (%)* | | 107 (6·2) | 13 (18·1) | 10 (10·8) | <0·001 |  |
|  |  | |  | | | | | |
| **Medication** | **Levodopa Equivalent dose, mg** | | | 290·20 (4·6) | 281·23 (23·1) | 348·34 (20·4) | 0·016^b^ |  |
|  |  | *Untreated, n (%)* | |  |  |  |  |  |

^a^ Difference between PD-T2DM and PD

^b^ Difference between PD-T2DM and PD-T2DM/Met

^c^ Difference between PD and PD-T2DM/Met

MCI (Mild cognitive impairment); ICD (impulse control disorder); MDS-UPDRS (Movement Disorders Society Parkinson’s Disease Rating scale); H&Y (Hoehn & Yahr stage)

**Supplementary Figure 4:**

Longitudinal impact of T2DM on symptoms in PD per group. Multivariate Cox regression analyses for the development of each disease marker / clinical outcome in PD according to the presence of T2DM. Multivariate analyses adjusted for age, sex, ethnicity, disease duration, vascular score, Hoehn & Yahr stage, BMI and LEDD.


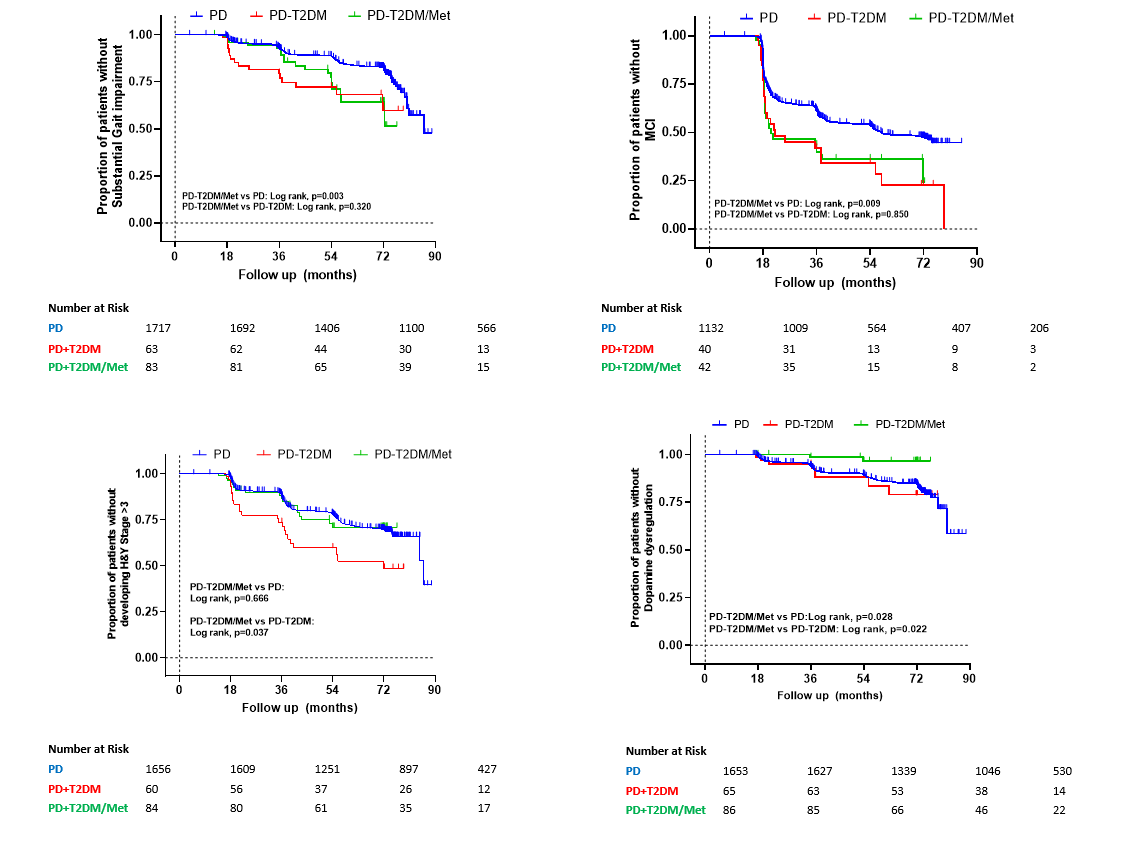


**Supplementary Figure 5:**


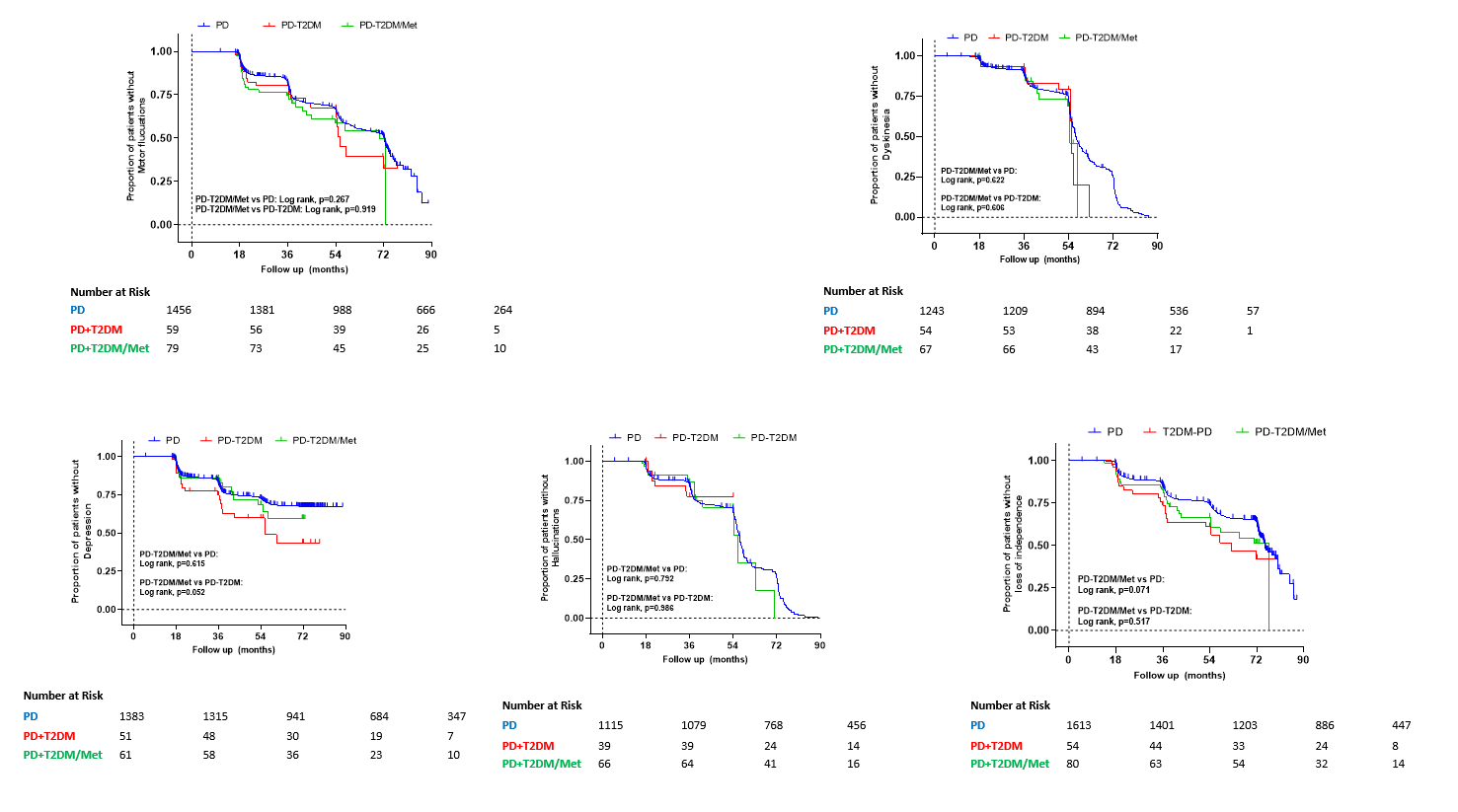
Longitudinal impact of T2DM on symptoms in PD per group.

**Supplementary Table 3:**

Multivariate Cox regression analyses for the development of each disease marker / clinical outcome in patients with PD according to the use of metformin. Multivariate analyses adjusted for age, sex, vascular score, ethnicity, disease duration, Hoehn & Yahr stage, BMI and LED

| Disease marker / clinical milestone | PD-T2DM/Met  vs  PD-T2DM | | PD-T2DM/Met  Vs  PD | |
| --- | --- | --- | --- | --- |
|  | Multivariate  HR (95% CI) | p value | Multivariate  HR (95% CI) | p value |
| **Substantial gait impairment**  *(MDS-UPDRS 3·10>3)* | 1·06 (0·48-2·34) | 0·890 | 1·212 (0·94-1·56) | 0·137 |
| **MCI**  *(MoCA<26)* | 0·91 (0·49-1·68) | 0·768 | 1·18 (0·96-1·45) | 0·112 |
| **Development of H&Y >3** | 0·62 (0·31-1·20) | 0·157 | 0·89 (0·70-1·13) | 0·370 |
| **Hallucinations**  *(MDS-UPDRS 1·2>1)* | 0·70 (0·23-2·15) | 0·538 | 0·98 (0·74-1·29) | 0·908 |
| **Depression**  *(LADS>6)* | 1·34 (0·64-2·82) | 0·426 | 1·34 (1·06-1·71) | 0·015 |
| **ICD**  (QUIP 1-4 >1) | 0·65 (0·30-1·39) | 0·266 | 0·97 (0·75-1·25) | 0·833 |
| **Dyskinesia**  *(MDS-UPDRS 4·1>1)* | 1·32 (0·38-4·53) | 0·654 | 1·05 (0·76-1·43) | 0·740 |
| **Motor fluctuations** | 1·17 (0·59-2·30) | 0·654 | 1·12 (0·91-1·38) | 0·260 |
| **Loss of independence**  *(SE-ADL<80%)* | 1·18 (0·93-3·53) | 0·080 | 1·08 (0·89-1·32) | 0·406 |

**Supplementary Figure 6:**

Linear mixed modelling progression of symptoms of PD vs PD+T2DM and PD+T2DM/Met


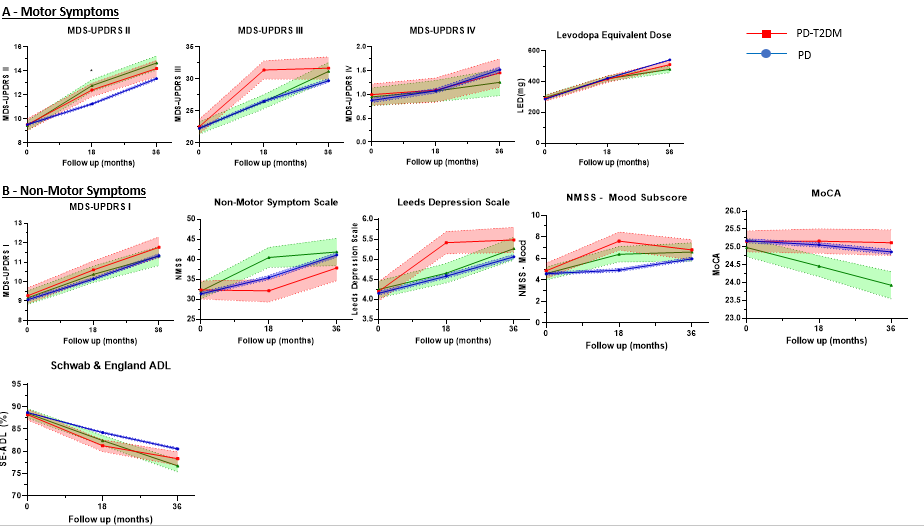


Sensitivity analysis: Does the increase in vascular disease drive the effects of T2DM on PD disease progression?

Models were repeated using vascular score as the dependent variable, to compare the effects of vascular disease versus T2DM on aspects of disease progression (Supplementary Table 4 &5). All models were adjusted for differences in age, sex, ethnicity, disease duration, LEDD, H&Y Stage, and p values were adjusted for multiple comparisons (Supplementary Data). In the subgroup of PD patients without T2DM but with additional vascular diseases (PD/VasR+, n=906), there was no significant worsening of PD disease severity or progression compared to PD patients without T2DM and no vascular risk disease (PD/VasR-, n=857). However, when PD patients with T2DM (PD+T2DM, n=167) were compared to patients with increased vascular disease (PD/VAsR+, n=906), the PD+T2DM group had significantly worse symptoms based on non-motor scores (NMSS Total: 7.1 points (0.9-13.2) p=0.017; UPDRS I: 1.5 points (0.4-2.6), p=0.003; Leeds Depression Index: 0.68 points (0.02-1.33), p=0.039); Sleep scores (PDSS -7.1 points (-12.0-2.2), p=0.002; ESS 0.93 points (0.01-1.86), p=0.045); cognition (MoCA -1.3 points (-2.0—0.6), p=<0.0001); and quality of life scores (EQ5D VAS -5.3 points (-8.7—1.9), p=0.001, SE-ADL -3.3 points (-5.4—1.1), p=0.001). There were significant differences in motor scores between patient with T2DM and PD patients with no vascular risk (data not shown) but no statistical differences in motor scores between the T2DM group and PD/VasR.

**Supplementary Table 4:**

Clinical Features of patients within 18 months of diagnosis of PD. Patients were categorised into patients with no T2DM and no vascular risk factors (PD/VasR-), patient with no T2DM but increased vascular risk factors (PD/VasR+), and patients with co-morbid T2DM (PD+T2DM). There were no significant differences between the PD/VasR- and PD/VasR+ groups, but when compared to the increased vascular risk factors group (without T2DM), patients with T2DM had significantly worse scores in depression, quality of life and substantial gait impairment.

|  |  | | PD/VasR-  N= 857 (44.4%) | PD/VasR+  N=906 (46.9%) | p value*^a^ | PD+T2DM  N=167 (8.7%) | P value^*b^ |
| --- | --- | --- | --- | --- | --- | --- | --- |
| **Demographics** | Age | | 67.4 (0.3) | 66.9 (0.3) | 0.907 | 71·1 (0·7) | <0.0001 |
|  | Age of diagnosis | | 66.1 (0.3) | 65.7 (0.3) | 1.000 | 69·7 (0·6) | <0.0001 |
|  | Disease duration, years | | 1.37 (0.1) | 1.30 (0.1) | 0.372 | 1.33 (0.1) | 1.000 |
|  | Sex, males (%) | | 552 (64.4) | 5858 (64.6) | 1.000 | 121 (72·5) |  |
|  | BMI | | 26.7 (0.2) | 26.8 (0.1) | 1.000 | 29·7 (0·4) | <0.0001 |
| **Aspect of PD** | **Scale** | |  |  |  |  |  |
| **Non-motor symptoms** | UPDRS I | | 9.05 (0.2) | 9.329 (0.2) | 1.000 | 10.82 (0.4) | 0.003 |
|  | NMSS Total | | 30.82 (1.0) | 32.58 (1.0) | 0.641 | 39.7 (2.3) | 0.017 |
|  | Leeds Anxiety Index | | 3.97 (0.1) | 4.23 (0.1) | 0.407 | 4.62 (0.2) | 0.598 |
|  | **Leeds Depression Index** | | **4.12 (0.1)** | **4.33 (0.1)** | **0.541** | **5.00 (0.3)** | **0.039** |
| **Sleep** | **PDSS** | | **111.67 (0.8)** | **109.14 (0.8)** | **0.086** | **102.01 (1.9)** | **0.002** |
|  | ESS | | 6.78 (0.2) | 6.79 (0.2) | 0.218 | 7.73 (0.4) | 0.045 |
| **Cognition** | MoCA total | | 25.01 (0.1) | 24.94 (0.1) | 1.000 | 23.60 (0.3) | <0.0001 |
| **Motor features** | UPDRS II | | 9.70 (0.2) | 9.89 (0.2) | 1.000 | 10.3 (0.5) | 1.000 |
|  | UPDRS III | | 22.70 (0.3) | 22.9 (0.4) | 1.000 | 24.56 (0.8) | 0.219 |
|  |  | ***Substantial gait impairment, n (%)*** | ***24 (2.8)*** | ***2.4 (2.4)*** | ***0.723*** | ***20 (12.1)*** | ***<0.0001*** |
|  | UPDRS IV | | 0.76 (0.1) | 0.70 (0.1) | 1.000 | 0.82 (0.1) | 1.000 |
| **Quality of life** | PDQ8 total | | 5.63 (0.2) | 5.92 (0.2) | 0.585 | 6.37 (0.4) | 0.789 |
|  | EQ5D VAS | | **77.67 (0.6)** | **76.90 (0.5)** | **0.989** | **71.59 (1.3)** | **0.001** |
|  | EQ5D Index | | **0.74 (0.01)** | **0.73 (0.01)** | **1.000** | **0.67 (0.01)** | **<0.0001** |
|  | SE-ADL | | **88.51 (0.4)** | **88.58 (0.3)** | **1.000** | **85.27 (0.8)** | **0.001** |
| **Medication** | Levodopa Equivalent daily dose, mg | | **288.79 (6.7)** | **292.49 (6.4)** | **1.000** | **333.06 (15..0)** | **0.040** |

^a^PDVasR- vs PDVasR+; ^b^PD+T2Dm vs PD/VasR+; p-values adjusted for multiple comparisons (Bonferroni)

**Supplementary Figure 7:**


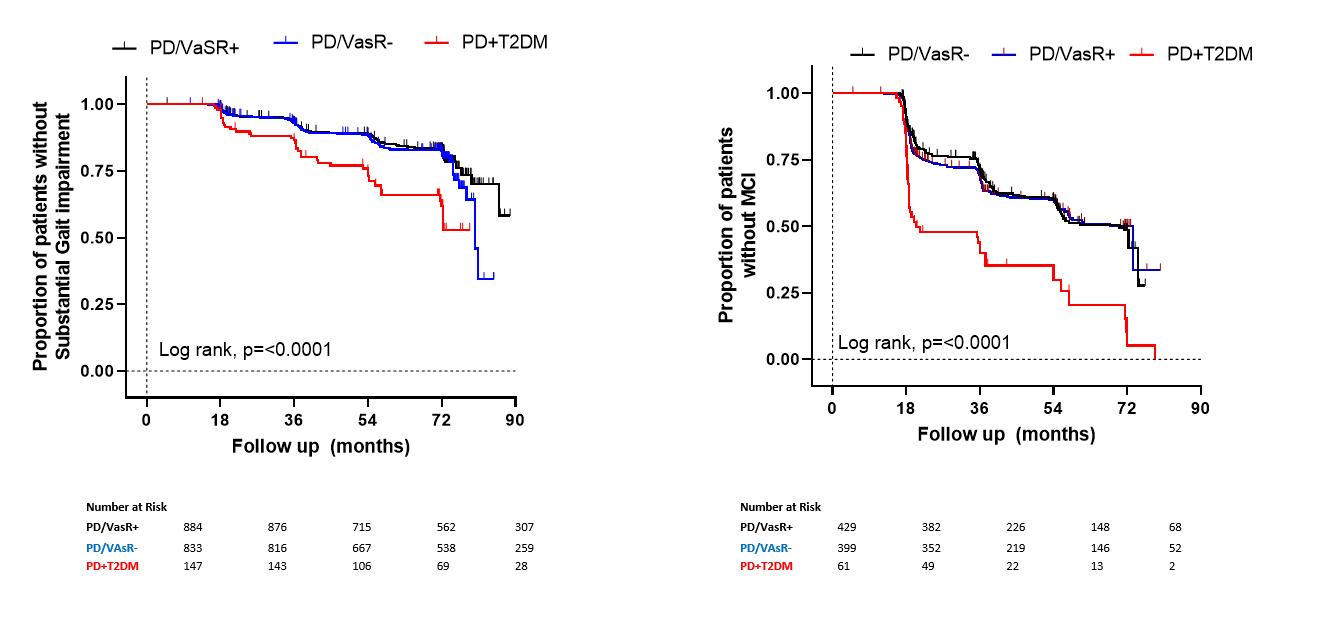
Longitudinal follow up. Univariate regression analyses for the development of each disease marker / clinical outcome in PD according to the presence of no vascular risk factors (PD/VasR-), increased vascular risk factors (PD/VasR+), and T2DM.

**Supplementary Table 5:**

Multivariate Cox regression analyses for the development of each disease marker / clinical outcome in patients with PD according to the use of metformin. Multivariate analyses adjusted for age, sex, ethnicity, disease duration, Hoehn & Yahr stage, BMI and LED

| **Disease marker / clinical milestone** | **PD**  **vs**  **PD/VasR** | | **PD+T2DM**  **Vs**  **PD/VasR** | |
| --- | --- | --- | --- | --- |
|  | Multivariate  HR (95% CI) | p value | Multivariate  HR (95% CI) | p value |
| **Substantial gait impairment** *(MDS-UPDRS 3·10>3)* | 1.04 (0.8 – 1.4) | 0.763 | 1.59 (1.1 – 2.4) | 0.024 |
| **MCI** *(MoCA<26)* | 0.952 (0.7 – 1.2) | 0.687 | 1.45 (0.9 – 2.2) | 0.069 |

## Data sharing

The data that support the findings of this study are available on request from the *Tracking-PD* committee.

**References:**

1 Ou R, Wei Q, Hou Y, *et al.* Effect of diabetes control status on the progression of Parkinson’s disease: A prospective study. *Ann Clin Transl Neurol* 2021; **8**: 887–897.

2 Pagano G, Polychronis S, Wilson H, *et al.* Diabetes mellitus and Parkinson disease. *Neurology* 2018; **90**: e1654–e1662.

3 Mollenhauer B, Zimmermann J, Sixel-Döring F, *et al.* Baseline predictors for progression 4 years after Parkinson’s disease diagnosis in the De Novo Parkinson Cohort (DeNoPa). *Mov Disord* 2019; **34**: 67–77.

4 Mohamed Ibrahim N, Ramli R, Koya Kutty S, Shah SA. Earlier onset of motor complications in Parkinson’s patients with comorbid diabetes mellitus. *Mov Disord* 2018; **33**: 1967–1968.

5 Prange S, Danaila T, Laurencin C, *et al.* Age and time course of long-term motor and nonmotor complications in Parkinson disease. *Neurology* 2019; **92**: e148–e160.

6 Litvan I, Goldman JG, Tröster AI, *et al.* Diagnostic criteria for mild cognitive impairment in Parkinson’s disease: Movement Disorder Society Task Force guidelines. *Mov Disord* 2012; **27**: 349–356.

7 Hoogland J, Boel JA, de Bie RMA, *et al.* Risk of Parkinson’s disease dementia related to level I MDS PD-MCI. *Mov Disord* 2019; **34**: 430–435.

8 Hoops S, Nazem S, Siderowf AD, *et al.* Validity of the MoCA and MMSE in the detection of MCI and dementia in Parkinson disease. *Neurology* 2009; **73**: 1738–1745.

9 Kim HM, Nazor C, Zabetian CP, *et al.* Prediction of cognitive progression in Parkinson’s disease using three cognitive screening measures. *Clinical Parkinsonism & Related Disorders* 2019; **1**: 91–97.

10 Bowling A, Gabriel Z, Dykes J, *et al.* Let’s ask them: a national survey of definitions of quality of life and its enhancement among people aged 65 and over. *Int J Aging Hum Dev* 2003; **56**: 269–306.

11 Evans JR, Mason SL, Williams-Gray CH, *et al.* The natural history of treated Parkinson’s disease in an incident, community based cohort. *J Neurol Neurosurg Psychiatry* 2011; **82**: 1112–1118.

12 McRae C, Diem G, Vo A, O’Brien C, Seeberger L. Reliability of measurements of patient health status: a comparison of physician, patient, and caregiver ratings. *Parkinsonism Relat Disord* 2002; **8**: 187–192.

13 Weintraub D, Hoops S, Shea JA, *et al.* Validation of the questionnaire for impulsive-compulsive disorders in Parkinson’s disease. *Mov Disord* 2009; **24**: 1461–1467.

14 Snaith RP, Bridge GW, Hamilton M. The Leeds scales for the self-assessment of anxiety and depression. *Br J Psychiatry* 1976; **128**: 156–165.

15 Weng H-Y, Hsueh Y-H, Messam LLM, Hertz-Picciotto I. Methods of covariate selection: directed acyclic graphs and the change-in-estimate procedure. *Am J Epidemiol* 2009; **169**: 1182–1190.

16 Tennant PWG, Murray EJ, Arnold KF, *et al.* Use of directed acyclic graphs (DAGs) to identify confounders in applied health research: review and recommendations. *Int J Epidemiol* 2021; **50**: 620–632.

17 Shrier I, Platt RW. Reducing bias through directed acyclic graphs. *BMC Med Res Methodol* 2008; **8**: 70.

18 Textor J, van der Zander B, Gilthorpe MS, Liskiewicz M, Ellison GT. Robust causal inference using directed acyclic graphs: the R package “dagitty”. *Int J Epidemiol* 2016; **45**: 1887–1894.
